# Development and Implementation of the *How to Talk to Your Doctor HANDbook* Health Literacy Program in Rural Counties

**DOI:** 10.3928/24748307-20190731-01

**Published:** 2019-09-06

**Authors:** Lisa Washburn, Kristie B. Hadden, Latrina Y. Prince, Charleen McNeill, Zola Moon

## Abstract

**Background:**

Improvements in health literacy are unlikely without intervention in community settings. However, interventions appropriate for delivery in these settings are lacking, limiting reach to rural adults who are disproportionately affected by low health literacy and poor health outcomes. The *How to Talk to Your Doctor (HTTTYD) HANDbook* Program was developed through a research-practice partnership to educate rural residents to effectively advocate and participate in their own health care.

**Brief Description of Activity:**

We describe development of the *HTTTYD HANDbook* Program delivered through the Cooperative Extension Service to educate adults who are eligible for Medicaid and have low health literacy. *HTTTYD HANDbook* implementation is described using the RE-AIM (reach, effectiveness, adoption, implementation, and maintenance) framework (and specifically the reach, adoption, implementation, and maintenance dimensions).

**Implementation:**

The *HTTTYD HANDbook* was developed using health literacy best practices with user-centered design, and it was field tested with community members with varying levels of health literacy. Reach, adoption, implementation, and maintenance of the *HTTTYD HANDbook* were assessed by tracking distribution of *HTTTYD HANDbook* Program materials, return submission of evaluation and tracking instruments, adherence to program and data collection/submission protocols, and program continuation.

**Results:**

Overall reach into the population was 6 per 10,000; about 25% were Medicaid recipients and 28.2% had low health literacy. Most participants were age 65 years or older. Of the 72 counties with program access, 52.7% requested *HTTTYD HANDbook* Program materials; 31% adopted the program, but only 30% of these counties adhered to program implementation and data collection protocols. Reach and adoption were higher among rural counties, and rural counties were more likely than nonrural counties to maintain the *HTTTYD HANDbook* Program.

**Lessons Learned:**

The *HTTTYD HANDbook* Program addresses barriers to engagement in patient-provider communication for rural, low-income community members. Programs can be implemented in community settings through established local organizations, such as county extension offices, to increase access for rural adults. Implementation barriers included staff turnover and transportation of program materials. Online facilitator training availability had little impact on adherence to program protocols. Organizational context and established procedures for program delivery and evaluation should be considered in adoption decisions and integrated into implementation protocols. **[*HLRP: Health Literacy Research and Practice*. 2019;3(3):e205–e215.]**

**Plain Language Summary:**

The *How to Talk to Your Doctor HANDbook* Program was created with people from the community to help patients prepare for doctor visits. The *How to Talk to Your Doctor HANDbook* Program helps patients to overcome barriers to talking to their doctor so that they can better understand how to get healthy and stay healthy.

Rural residents are disproportionally affected by low health literacy, further compounding the poorer health outcomes experienced by this population. Health literacy, defined as the degree to which people have the capacity to obtain, process and understand basic health information and services needed to make appropriate health decisions (Parker, Ratzan, & Lurie, 2003), is linked to educational attainment and socioeconomic status. In addition to rural residents, African Americans and other racial and ethnic minorities, people with less than a high school education, and those who live in poverty are more likely to have low levels of health literacy (Arkansas Department of Health, 2013). Low health literacy may lead to poorer health outcomes and higher health care costs, particularly for the medically fragile and those managing a chronic disease (Zahnd, Scaife, & Francis, 2009). Improved health literacy is necessary for efforts to improve health care quality, reduce costs, and reduce disparities to succeed (Kindig, Panzer, & Nielsen-Bohlman, 2004).

Improvements in health literacy are unlikely without intervention in community settings. Programs to address these issues in populations with low health literacy are needed, particularly in rural areas where residents are poorer and less healthy than their urban counterparts (Arkansas Department of Health, 2013). The Cooperative Extension System, a nationwide network of nonformal county-based educators associated with the land-grant university system, is uniquely positioned to provide outreach and education aimed at building health literacy skills. Extension professionals have long addressed local health issues through education and outreach, but it was not until 2014 that the National Framework for Health and Wellness was established, which stated that “the same system of Extension can do for the nation's health what it did for American agriculture” (Braun et al., 2014). This Framework includes health literacy as one of seven major focus areas to address public health (Braun et al., 2014). County Extension educators (hereafter termed “health educators”) provide local education in community settings.

## Background

To improve individual and family health literacy skills around the domain of communication with health care providers for Medicaid-eligible Arkansans residing in rural areas, the *How to Talk to Your Doctor* (*HTTTYD*) *HANDbook* Program was developed through a research-practice partnership and delivered through the Cooperative Extension Service in one state. Given the lack of health literacy interventions developed for delivery by health educators in community settings, the *HTTTYD HANDbook* fills an important gap. This article describes the program development process and implementation outcomes guided by the RE-AIM (reach, effectiveness, adoption, implementation, and maintenance) framework (Glasgow, 2002; Kessler et al., 2013).

### Program Background and Purpose

The overall purpose of the *HTTTYD HANDbook* Program was to educate Arkansans with limited resources and low health literacy so they can effectively advocate and participate in their own health care, ultimately improving health for themselves and their families. The National Cooperative Extension Framework for Health and Wellness (Braun et al., 2014) was the guiding framework for the project, which aimed to improve health among rural Arkansas residents who had low income by increasing knowledge and confidence, and through a tailored approach to improve health literacy skills with a focus on health communication for medically vulnerable populations.

### Program Development

**Figure 1.**
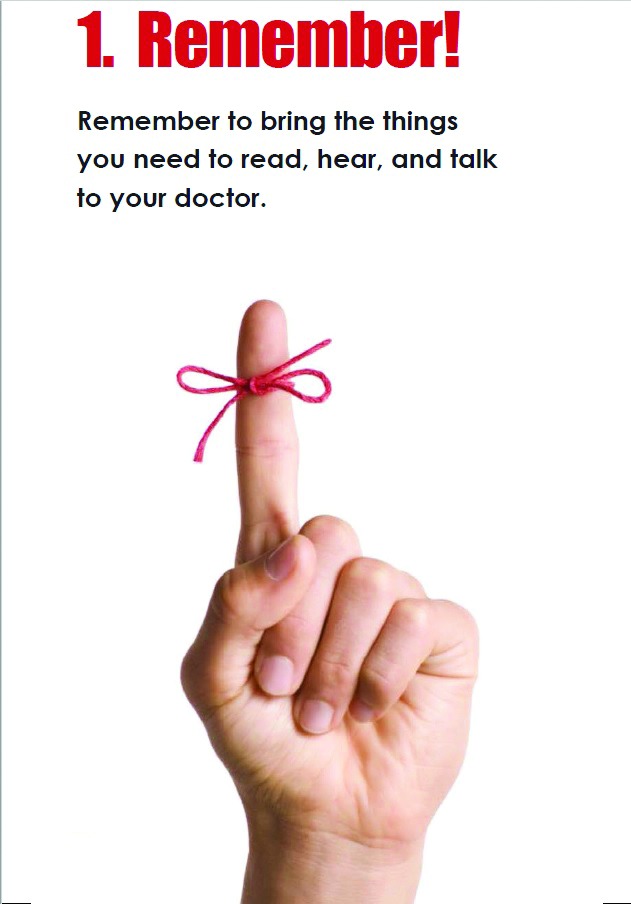
*How to Talk to Your Doctor HANDbook*, Step 1: Remember!

A review of existing health literacy programs and materials offered through the land-grant extension system revealed a lack of suitable materials for use in community settings with rural, low-income audiences with low health literacy. Thus, our approach led to development of new materials employing non–literacy-dependent strategies and user-centered design. The non–literacy-dependent strategies included using fewer words, more graphics, and including the person's hand as a tool to remember content so that reading is less essential to learning and retaining (**Figure 1**). These strategies were based on the Zimbabwe Hand Jive (Canadian Diabetes Association, 2003), which uses a person's hand instead of written materials to learn and remember dietary guidelines for nonliterate cultures all over the world. This approach was selected because we recognized that many written resources, regardless of how well they are written and designed, may not meet the needs of community members with low literacy.

The project was initiated as an expansion of an existing workbook (Brown, 2013) but shifted to a new approach after review of evidence and community input. Review of research and practice literature revealed 13 existing tools (Agency for Healthcare Research and Quality, 2011a; Agency for Healthcare Research and Quality, 2011b; Agency for Healthcare Research and Quality, 2011c; Agency for Healthcare Research and Quality, 2014; National Institute on Aging, 2014a; National Institute on Aging, 2014b; National Institute on Aging, 2014c; National Institute on Aging, 2014d; National Institute on Aging, 2014e; National Institute on Aging, 2014f; National Institute of Diabetes and Digestive and Kidney Diseases, 2013; National Women's Health Information Center, 2008; Shojania, Duncan, McDonald, Wachter, & Markowitz, 2001; The Conversation Project & Institute of Healthcare Improvement, 2013) that aimed to improve patient-provider communication for target audiences similar to ours. Content analysis revealed 17 unique constructs related to patient-provider communication in these materials. Frequency of occurrence of each construct was tabulated and shared with a panel of six health literacy experts and four physicians. The expert group vetted the list of constructs and ranked the top six constructs based on expert opinion, strength of evidence, and frequency of occurrence. The top six constructs noted in **Table 1** were included in the first prototype of the new *HTTTYD HANDbook*.

Field testing of the new *HTTTYD HANDbook* with community members with varying levels of health literacy informed changes in content, organization, and style for the second prototype. A new tool prototype was designed using health literacy and plain language best practices (Centers for Disease Control and Prevention, 2015; Centers for Medicare and Medicaid Services, 2016; National Institutes of Health, 2013). Input from a focus group of eight community members with varying levels of health literacy provided guidance on the following: title, organization of information, understandability of content, actionability of content, and style preferences. A second prototype was created based on focus group feedback, field tested again, and similar changes were made to the final product, the *HTTTYD HANDbook*. Readability assessment was conducted using three formulas (Flesch, 1948; Fry, 1977; McLaughlin, 1969) prior to finalizing each prototype, and plain language editing ensured that the reading level did not exceed fifth grade. Readability assessment of the final version revealed a mean level of fourth grade (Flesch, 1948; Fry, 1977; McLaughlin, 1969). Project protocol was approved by the University of Arkansas Institutional Review Board.

## Brief Description of Activity

The *HTTYD HANDbook* Program is a single-session program designed for delivery in community-based settings. The program was piloted by extension health educators but is appropriate for delivery by others in community settings. Sessions aimed to teach rural residents of Arkansas strategies for improving communication with health care providers. *HTTTYD HANDbook* sessions used the *HTTTYD HANDbook* as the primary teaching tool. Teaching techniques used modeling, scenarios, role-play, and Teach Back, which provided participants opportunities to practice new communication skills. Examples of recommended strategies included making a list of questions to take to appointments, writing down prescriptions prior to provider visits, writing down reasons for the visit and changes in health status in preparation for appointment, taking written notes during the visit, and requesting written instructions for medicine and treatments. Sessions typically lasted 1 hour. Participants received the *HTTTYD HANDbook* and a Med HANDbag (insulated lunch tote imprinted with program artwork) for transporting medicines to their health care provider appointments.

## Implementation

### Program Implementation

***Targeted counties and population.*** The *HTTTYD HANDbook* program was available to 72 Arkansas counties through the county extension health educator. Program availability was promoted through a webinar introducing program components and protocol, presentation at a statewide conference attended by health educators, and through emails to the health educator distribution list. Training to become a web-based facilitator was available. This web-based training featured a 30-minute video addressing best practices for teaching low-literacy audiences in community settings and modeled the *HTTTYD HANDbook* session step-by-step. Because of limited Internet access for some rural areas, a downloadable lesson guide for the session including roughly the same content (and data collection protocol) was available for those teaching *HTTTYD HANDbook* sessions but who may not have completed the facilitator training. Training was not required to conduct *HTTTYD HANDbook* sessions. The health educators, as nonformal educators, were generally knowledgeable of *HTTTYD HANDbook* content and accustomed to delivering community-based programs (Monroe et al., 2005).

Health educators obtained *HTTTYD HANDbook* materials (i.e., the *How to Talk to Your Doctor HANDbook*, Med HANDbag, and copies of data collection instruments) by contacting the state *HTTTYD HANDbook* coordinator. Materials were provided at no cost to health educators, and *HTTTYD HANDbook* sessions were free to participants. All material requests and quantities provided were logged, as well as return of data collection instruments as instructed in the program protocol. *HTTTYD HANDbook* materials were available for request from August 2016 through September 2017.

Rural adult Arkansans and Medicaid recipients were a target audience for *HTTTYD HANDbook* sessions, but participation was not limited to this group; sessions were open to all adults age 18 years and older. County health educators were provided with recruitment materials, including a news article, flier, and sample social media post. *HTTTYD HANDbook* Program delivery sites were locally determined based on the county health educator's knowledge of the community and program opportunities.

***Data collection.*** Rural-Urban Continuum codes were used to categorize counties as rural (codes 4–9) or nonrural (codes 1–3) (United States Department of Agriculture Economic Research Service, 2013). Counties requesting *HTTTYD HANDbook* materials and returning participant data within the data collection timeframe ending December 2017 were defined as implementing counties. For the purpose of this article, *HTTTYD HANDbook* participants were defined as those for whom complete data were returned after involvement in a *HTTTYD HANDbook* session. Counties requesting *HTTTYD HANDbook* materials but that did not return data where considered as intending to adopt.

### RE-AIM and Evaluation Aims

The RE-AIM framework was used to evaluate *HTTTYD HANDbook* implementation. The RE-AIM framework includes five dimensions: (1) reach (participation rate and representativeness of participants), (2) effectiveness (impact on outcomes), (3) adoption (participation at the setting level [i.e., county within an extension context] and staff level [i.e., health educators]), (4) implementation (consistency of delivery and adherence to program protocols), and (5) maintenance (continuing to offer the program over time). Reach, adoption, implementation, and maintenance dimensions are described here and in **Table 2** (Glasgow, 2003). *HTTTYD HANDbook* effectiveness is reported elsewhere (McNeill, Washburn, Hadden, & Moon, 2019). RE-AIM is useful in determining factors contributing to program success in real-world settings, which is important for broad dissemination of evidence-based programs (Green & Glasgow, 2006). This framework has been used to guide implementation evaluation of rural family caregiver education programs (Samia, Aboueissa, Halloran, & Hepburn, 2014), national dissemination of a community-based heart health program (Folta et al., 2015a; Folta et al., 2015b), and to systematically review published health literacy interventions (Allen, Zoellner, Motley, & Estabrooks, 2011).

***RE-AIM components.*** *Reach and representativeness*. Participants providing informed consent also provided demographic information (age, race/ethnicity, Medicaid recipient status). Overall reach for counties implementing *HTTTYD HANDbook* was calculated by dividing the number participants (those for whom complete data were received) by total population age 18 years and older in implementing counties. Reach to adults age 65 years and older was calculated similarly, by dividing the number of participants age 65 years and older by the county population age 65 years and older. After calculating reach for the project overall, reach for rural and nonrural implementing counties was computed.

Representativeness of participants in terms of Medicaid recipient status was calculated by comparing the percent of participants eligible for Medicaid with the percent of adult population eligible in implementing counties. Health educators reported information on number of participants, county, and class location (i.e., community center, church, senior center) for each session conducted using a report form returned with hard copies of participant data. Participants completed a questionnaire at the start of the *HTTTYD HANDbook* session including a health literacy screening question, “How confident are you filling out medical forms by yourself?” (Stagliano & Wallace, 2013). Responses to this question were used to determine the proportion of participants who have inadequate health literacy.

*Adoption.* The overall adoption rate at the setting-level was calculated as the proportion of eligible counties conducting *HTTTYD HANDbook* sessions (i.e., the number of counties with a health educator position). We define setting, within the context of extension program delivery, as the county. Representativeness of settings was also considered by comparing adoption by rural and nonrural counties. We also considered intent to adopt, calculated as the number of eligible counties requesting *HTTTYD HANDbook* materials.

Adoption at the staff level focused on impact of the online facilitator training. Staff were defined as extension health educators conducting *HTTTYD HANDbook* sessions. Completion of online training was not required to conduct sessions, thus educators fell into one of three groups: (1) those who both completed the online training and conducted sessions, (2) those completing the online training but failing to conduct *HTTTYD* sessions, and (3) those who did not complete the online training but conducted sessions. Adoption at the staff level was calculated based on the number of educators completing the online facilitator training who also conducted sessions following implementation protocols. We also considered the proportion of educators who conducted *HTTTYD HANDbook* sessions but did not complete the facilitator training and rural or nonrural status.

*Implementation.* Requests for *HTTTYD HANDbook* materials and return of participant, informed consent, and evaluation data were logged. Implementation was calculated based on the proportion of counties requesting *HTTTYD HANDbook* materials that also conducted sessions (i.e., adopting counties) that adhered to the data collection and return protocol and timeframe. Health educators in counties receiving materials but not submitting data within the data collection timeframe were contacted to determine implementation status. We also determined use of materials requested by rural and nonrural counties, defined as the number of participants for whom data were returned divided by the quantity of materials requested.

*Maintenance.* Maintenance of *HTTTYD HANDbook* among adopting counties was calculated as the proportion of implementing counties where *HTTTYD HANDbook* sessions were conducted more than one time within the implementation timeframe.

## Results

**Figure 2.**
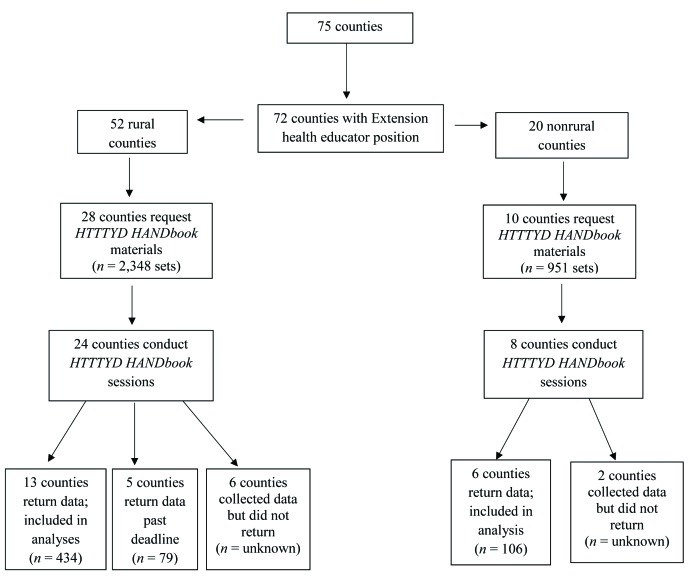
The *How to Talk to Your Doctor (HTTTDY) HANDbook* Program implementation in rural and nonrural counties.

Of 72 counties with program access, 52 were rural and 20 were nonrural. Thirty-eight counties requested and were provided with *HTTTYD HANDbook* materials (*HTTTYD HANDbook* and Med HANDbag) and copies of data collection instructions and instruments (informed consent forms, pre-/post-class questions). Of those, 19 counties had data included in analyses (participant demographics, pre- and post-class questions). Educators in 13 counties who received materials but did not submit data were contacted by email to determine implementation status; 11 educators responded. Of these, five health educators had conducted the program but returned paperwork outside the data analysis timeframe, eight reported conducting the program but neglected to return paperwork, and one health educator reported they had not conducted the program but intend to within the next 6 months. Because of health educator vacancies (*n* = 2) and nonresponse to follow-up (*n* = 1), information for three counties could not be collected (**Figure 2**).

### Reach

A total of 548 adults in 32 counties participated in *HTTTYD HANDbook* sessions. The majority were White (65.9%), female (83.8%), age 65 years and older (65.9%), and were from rural counties (76%). One-quarter were Medicaid recipients. Across all implementing counties, reach (or the proportion of eligible adult participants) was 0.06% overall and 0.21% for those age 65 years and older. Reach was higher among rural counties, where overall reach was 0.19%, and reach to those age 65 years and older was 0.54%. Across all counties returning data within the implementation time-frame, 25% of participants were Medicaid recipients; the proportion of Medicaid recipients was higher in nonrural counties (32%) than in rural counties (24%). Most *HTTTYD HANDbook* participants attended sessions in senior centers (39.5%) and churches (19.9%). Twenty-eight percent of participants screened were identified as having inadequate health literacy.

### Adoption

***Setting level.*** Of 72 eligible counties, 38 requested *HTTTYD HANDbook* materials, indicating adoption intentions among 52.7% of eligible counties (53.4% for rural, 50% for nonrural). Overall 31% (*n* = 32) of eligible counties adopted the *HTTTYD HANDbook* program (i.e., requested materials and conducted sessions). Adoption was higher among rural counties; 46% of rural counties (*n* = 24) adopted *HTTTYD HANDbook* compared with 40% of nonrural counties (*n* = 8).

***Staff level.*** Sixteen health educators completed the online training. Of these, 13 requested *HTTTYD HANDbook* materials and seven implemented the program, yielding an overall adoption rate of 43.7% among those completing the *HTTTYD HANDbook* facilitator training. Online training use among educators in adopting counties was 40.6%; educators in a majority of adopting counties opted to conduct sessions using only the printed lesson materials. Educators in adopting rural counties were less likely to complete online training; 23% of rural educators (*n* = 3) used the online training resource, compared with 83.3% of nonrural educators (*n* = 5).

### Implementation

Of the 38 counties requesting *HTTTYD HANDbook* materials, 32 reported conducting *HTTTYD HANDbook* sessions but only 19 submitted participant data within the data collection timeframe, yielding an implementation rate of 50% among counties requesting materials (i.e., those indicating intent to adopt). Of the 50% of counties requesting materials but not implementing as intended, health educators in three counties reported they did not conduct sessions, two were unavailable for follow-up due to health educator position vacancies, and one did not respond to follow-up requests. Five counties (all rural) reported conducting sessions but returned participant data outside the data collection timeframe. Eight counties (6 rural, 2 nonrural), or 21% of counties receiving materials, reported conducting *HTTTYD HANDbook* sessions and collecting but not submitting evaluation data, indicating failure to adhere to the program protocol. Counties used 30.5% of the 3,299 sets of *HTTTYD HANDbook* materials distributed, with rural counties using requested materials to a greater extent (39%) than nonrural counties (27%).

### Maintenance

Of implementing counties (*n* = 19), 57.8% (*n* = 11) conducted two or more *HTTTYD HANDbook* sessions indicating maintenance. Rural counties were more likely to maintain *HTTTYD HANDbook* (61.5% versus 50%) and conducted slightly more sessions overall (mean 3.875 sessions in rural counties versus 3.66 in nonrural counties).

## Lessons Learned

The RE-AIM Framework, which can help determine whether programs work in real-world settings, was used to evaluate *HTTTYD HANDbook* implementation (Glasgow, Vogt, & Boles, 1999). Given the need for community-based interventions addressing health literacy, these findings regarding reach, implementation, adoption, and maintenance provide valuable information for others seeking to adopt similar approaches for low literacy audiences in rural states.

Despite the ease of program delivery (i.e., single session, no equipment required) and flexibility for community locations, *HTTTYD HANDbook* was adopted by only 44.4% of counties (32 of 72 counties) with program access, and reach was lower than projected. One possible reason for lack of adoption in some counties is rates of turnover among county extension health educators. Newly hired educators may have been unaware of *HTTTYD HANDbook* availability or unclear on the process for training and requesting printed materials. Of eligible counties not requesting materials (*n* = 37), 16 counties, or 43%, experienced turnover during or just prior to the implementation period. Turnover among counties requesting materials was 31.5%; turnover among counties implementing *HTTTYD HANDbook* according to program protocols was 26%. Lengthy position vacancies are common and sometimes extend for more than 6 months. The program implementation period may not have allowed enough time for newly hired health educators to begin local program implementation after onboarding. Future *HTTTYD HANDbook* efforts should include plans for training and reaching out to new educators (Durlak & DuPre, 2008).

The impact of online *HTTTYD HANDbook* facilitator training availability and completion among educators is unclear. Among implementing counties (i.e., those conducting the program and following data submission protocols), most had health educators who did not complete the online training. In contrast, one-half of counties adopting the program but failing to comply with data collection and submission protocols had educators who completed the training. In the state where *HTTTYD HANDbook* was implemented, most programmatic training is conducted in a face-to-face fashion. The newness of online training, and availability of a detailed downloadable lesson guide as an alternative, may have influenced participation.

Program adopters rarely adopt or implement a program exactly as intended, and some flexibility may be desirable (Green & Glasgow, 2006). Eight counties (21%) reported conducting *HTTTYD HANDbook* sessions and collecting participant data but did not follow program protocols in returning data to the state coordinator. Low complexity and compatibility with organizational values enhance adoption and dissemination of interventions (Klesges, Estabrooks, Dzewaltowski, Bull, & Glasgow, 2005). In Arkansas where this study was conducted, extension program evaluation responsibilities and participant data typically reside at the county level; health educators are responsible for their own data aggregation, analysis, and reporting. Centralizing *HTTTYD HANDbook* data analysis diverged from usual procedures and possibly conflicted with an organizational value of health educator autonomy. Further, educators in this state are not routinely involved in research or data collection, presenting an additional issue for consideration when a high level of rigor is required. When health educators are unaccustomed to returning participant data to a centralized location, greater emphasis on following program protocols may be needed, particularly for seasoned educators who may follow well-worn processes for program implementation, focus primarily on program delivery, and fail to carry out important follow-up steps.

Counties requested far more *HTTTYD HANDbook* materials than were used. Participant data returned represented less than one-third of requested materials. However, actual rates of use are likely higher as counties conducting *HTTTYD HANDbook* sessions but not complying with data collection protocols are not included in this figure and counties “intending to adopt” reported utilization. Further, health educators may have requested materials with implementation plans beyond the data collection timeframe or may have intentionally overestimated the quantity needed to avoid travel to pick up additional materials, which were not shipped due to funding limitations.

## Study Limitations

One limitation of the program and evaluation reported here is the relatively short implementation timeframe, which does not allow for evaluation of program diffusion beyond early adopters. Additionally, we did not explore barriers to program implementation with nonadopters, so we are unable to describe barriers and potential solutions. Future work should examine barriers to implementation and explore effective strategies to engage community-based practitioners in evaluation standardized across sites. Health educators in this state are not routinely involved in research or data collection. Staff turnover and a lack of training or familiarity with evaluation processes may have presented an additional barrier. The method for transporting materials to counties may have also been a barrier. Arranging for transport of materials from extension headquarters to counties, as opposed to incurring shipping costs for bulky program materials, is routine organizational procedure. The potential for delay in making such arrangements may have prohibited “just in time” education for counties with program opportunities requiring quick turnaround and little planning time to acquire materials.

Although barriers to understanding the program materials were not identified, field testing was conducted with a focus group of participants from Central Arkansas, not from rural areas. In an effort to ensure the appropriate level of readability, understandability, and actionability, purposive sampling targeted participants with varying levels of self-reported health literacy rather than rurality.

## Conclusion

This work provides valuable information about development and implementation of community-based health literacy programs and illuminates potential implementation issues, particularly if rigorous data collection is planned in a decentralized system. Because of the national focus on health literacy, we anticipate that this educational program will be used by other state extension services to reach audiences with low health literacy. Given that decentralization and local autonomy are characteristic of local extension units across the nation, these findings are even more salient to ensure human and programmatic resources (i.e., printed materials) are used most efficiently.

## Figures and Tables

**Table 1:** Patient-Provider Communication Tools Review^a^: Constructs Found in Existing Tools and Identified by Experts

**Element/Construct/Skill Addressed in Existing Resources^a^**	**Frequency of Element/Construct/Skill Experts Identified (*N* = 10)**	**Cited Studies**
Ask questions^b^	9	Menendez et al., 2017; Galliher et al., 2010
Bring list of medications to show provider how you take your medications^b^	7	-
Get test results	3	-
Choosing health care facilities/providers	2	-
Understanding surgery	2	-
Informing providers about allergies	2	-
Understanding medication labels/instructions^b^	2	Bailey et al., 2014; Lee, Yu, You, & Son, 2017; Zarcadoolas, Vaughon, Czaja, Levy, & Rockoff, 2013
Provider safety measures (hand washing, surgery site confirmation)	1	-
Coordinating care with primary care provider	2	-
Sharing information with all providers	1	-
Ask trusted other to help give, receive, and/or understand information^b^	4	-
Accessing reliable, trusted information sources	1	-
Make and bring list of concerns	3	-
Giving accurate and updated information to providers^b^	5	-
Bringing glasses, hearing aids, other aids as needed to office visit or hospital	1	-
Accessing/using language services/interpreters	2	-
Repeating information in your own words^b^	1	Green, Gonzaga, Cohen, & Spagnoletti, 2014; Nouri & Rudd, 2015; White, Garbez, Carroll, Brinker, & Howie-Esquivel, 2013

Note.

a Agency for Healthcare Research and Quality, 2014; Agency for Healthcare Research and Quality, 2011a; Agency for Healthcare Research and Quality, 2011b; Agency for Healthcare Research and Quality, 2011c; The Conversation Project & Institute of Healthcare Improvement, 2013; National Institute on Aging, 2014a; National Institute on Aging, 2014; National Institute on Aging, 2014b; National Institute on Aging, 2014c; National Institute on Aging, 2014d; National Institute on Aging, 2014e; National Institute of Diabetes and Digestive and Kidney Diseases, 2013; National Women's Health Information Center, 2008.

b Priority element/construct/skill.

**Table 2:** RE-AIM Definitions and *How to Talk to Your Doctor HANDbook* Program Measures

**RE-AIM Dimension**	**Definition**	***How to Talk to Your Doctor HANDbook* Measures**
Reach	Participation rates and representativeness among intended audience	Proportion of eligible adults participating Proportion of participants with low health literacy Proportion of participants eligible for Medicaid
Adoption	Setting Extent of participation by counties Staff (educators) Extent of participation in online training component	Setting Percent of counties conducting sessions Representativeness of counties conducting sessions (rural vs. nonrural) Educators Percent completing online training who conducted sessions
Implementation	Extent to which program protocol delivered as intended	Percent of counties requesting materials that conducted sessions Percent of counties conducting sessions that followed data collection/submission protocol Percent of materials requested utilized
Maintenance	Setting level: program sustainability	Percent of counties implementing more than 1 session

Note. RE-AIM = reach, effectiveness, adoption, implementation, and maintenance.
